# Supplementary material for: The Effect of a Multifaceted Intervention on Dietary Quality in Schoolchildren and the Mediating Effect of Dietary Quality between Intervention and Changes in Adiposity Indicators: A Cluster Randomized Controlled Trial
Source: Nutrients. 2022 Aug 10;14(16):3272. doi: 10.3390/nu14163272 (PMC9414904; doi:10.3390/nu14163272)
Supplement: Supplementary file 1 [file nutrients-14-03272-s001.zip › nutrients-1808130-supplementary.pdf]

The Effect of a Multifaceted Intervention on Dietary Quality in Schoolchildren and the Mediating Effect of Dietary Quality between Intervention and Changes in Adiposity Indicators: A Cluster Randomized Controlled Trial

Jin-lang Lyu [1,2], Zheng Liu [1,2], Shuang Zhou [1,2], Xiang-Xian Feng [3], Yi Lin [4], Ai-Yu Gao [5], Fang Zhang [6], Li Li [7], Antje Hebestreit [8] and Hai-Jun Wang [1,2,*]

[1] Department of Maternal and Child Health, School of Public Health, Peking University, Beijing 100191, China
[2] National Health Commission Key Laboratory of Reproductive Health, Peking University, Beijing 100191, China
[3] Department of Preventive Medicine, Changzhi Medical College, Changzhi 046000, China
[4] Urumuqi Primary and Secondary School Health Care Center, Urumuqi 830000, China
[5] Dongcheng Primary and Secondary School Health Care Center, Beijing 100010, China
[6] Mentougou Primary and Secondary School Health Care Center, Beijing 102300, China
[7] Department of Endocrinology and Metabolism, Ningbo First Hospital, Ningbo 315000, China
[8] Leibniz Institute for Prevention Research and Epidemiology-BIPS, 28359 Bremen, Germany
* Correspondence: whjun@pku.edu.cn; Tel.: +86-10-82805583

Supplementary Table S1. Description of intervention components in the DECIDE-Children study.

Intervention components	Descriptions of the content	Implementation	Behavior change techniques
**Targeted to children**			
Health education activities	Key messages included the benefits of healthy weight, how to measure and assess weight and core methods of achieving a healthy weight (not eating excessively; not drinking sugar-sweetened beverage; eating less high-energy food; less sedentary behaviors; performing more physical activity) were systematically taught by trained teachers. Education books with instruction of healthy diet and "Nutrition evaluation turnplate for Chinese primary and middle school students" were delivered.	(1) Each school in the intervention group completed all the total of 10 activities (100%) and the attendance rate for each activity was above 99.9%<br>(2) Posters and slogan banners on campus were 100% delivered (a total of 4 times per school).	Instruction on how to perform the behavior, and goal setting,
Reinforcement of physical activity within school	At least one hour moderate-to-vigorous intensity physical activities at school per school day were guaranteed by trained physical education teachers.	Intervention schools achieved one hour of physical activity for a median of 83% school days. Among the three types of physical activities delivered, 88%, 84%, and 58% of physical education classes, extracurricular activities, and class-break exercise could achieve moderate-to-vigorous intensity, respectively.	Goal setting, habit formation and restructuring the physical environment.
Monitoring of children's weight and height	Each child's weight and height data were measured every month by trained teachers and input in into a software system for a smartphone app, from which children could get and view	(1) Each school in the intervention group completed all the total of 6 times (100%) of monthly monitoring. Children's attendance	Social support

	individualized feedbacks. Students' weight was monitored weekly by teacher in the classroom.	rates were above 90% all time points in all schools.<br>(2) Schools in the intervention group completed weekly monitoring for an average of 17.5 (range: 9-24) times.	
**Targeted to environment**			
Engaging schools to support	Health education activities and instructions regarding to the intervention were provided to school teachers. School policies (putting up health education posters and signs; not selling, eating, or buying unhealthy snacks or SSB within the school) were implemented to reduce intake. Curriculum time for health education and physical education was guaranteed at school.	(1) All schools (100%) in the intervention group implemented the policy of not selling unhealthy snacks or sugar-sweetened beverages within school, 66.7% (8/12) implemented the policy of not eating unhealthy snacks or sugar-sweetened beverages within school, and 66.7% (8/12) implemented the policy of not buying unhealthy snacks or sugar-sweetened beverages around school.<br>(2) All schools (100%) in the intervention group completed the health education as planned.	Restructuring the physical environment, social support and adding objects to the environment.
Engaging family to support	Three face-to-face health education sessions at the beginning and middle of the first semester and beginning of the second semester were delivered to parents. Parents were encouraged to promoted children's healthy diet and increase their physical activity. Parents were asked to record their children's BMI and behaviors	(1) Each school in the intervention group completed a total of 3 activities (100%) and the average attendance rate for each activity was 87.1% among all the intervention.<br>(2) Parental self-reported that 2.0% "never",	Self-monitoring of behavior, feedback on behavior, social support and review behavior goals.

related to lifestyles (such as diets and physical activity) weekly in the app and help their children to make behavioral progress based on the individualized feedbacks from researchers.	4.4% “very little”, 27.3% “sometimes”, 49.3% “often”, and 17.1% “always” encouraged children’s physical activity outside school.<br>(3) 99.7% of parents recorded behaviors of the children with a median of 25 times and 98.2% of parents tracked BMI of the children with a median of 14 times.

Supplementary Table S2. Scoring method of the revised DBI-07 for primary school children [a].

Components	Subgroup	Score	Scoring method
**C1-Cereals**	Cereals	-12~12	<75g=-12, (325~375) g=0, >625g=12; score decreased by 2 with intake amount decreased by 50g
**C2-Vegetables and fruits**	Vegetables	-6~0	≥400g=0, (200~399) g=-2, (1~199) g =-4, 0g=-6
	Fruits	-6~0	≥200g=0, (100~199) g=-2, (1~99) g =-4, 0g=-6
**C3-Milk and dairy products, Soybean and soybean product**	Milk and dairy products	-6~0	≥300g=0; score decreased by 1 with intake amount decreased by 50g
	Soybean and soybean products	-6~0	≥40g=0, (20~39) g=-2, (1~19) g=-4, 0g=-6
**C4-Animal foods**	Red meat, poultry and game	-4~4	0g=-4, (1~50) g=-2, (50~100) g=0, (101~150) g=2, >150g=4
	Fish and shrimp	-4~0	<20g=-4, (20~29) g=-3, (30~39) g=-2, (40~49) g=-1, ≥50g=0
	Eggs	-4~4	>75g=4, (51~75) g=2, (25~50) g=0, (1~24) g=-2, 0g=-4
**C5-Sugar-sweetened beverage and Unhealthy snacks [b]**	Sugar-sweetened beverage	0~6	0mL=0, (1~550) mL=3, >550mL=6
	Unhealthy snacks	0~6	(0~25) g=0, (26~50) g=3, >50g=6
**C6- Dietary diversity [c]**	Dietary diversity	-12~0	≥12 kinds of food (soybean is 5g) =0; score decreased by 1 with types of foods decreased by 1.
**C7- Drinking water**	Drinking water	-12~0	>1200mL=0, <100mL=-12, score decreased by 1 with intake amount decreased by 100mL

[a] Revised based on Chinese Dietary Guidelines and Food Guide Pagoda. [b] Replaced condiments and alcoholic beverage with sugar-sweetened beverage and unhealthy snacks.[c] 12 subgroups of dietary diversity score including: (1) rice and products; (2) wheat and products; (3) corn, coarse grains and products, starchy roots and products; (4) dark-colored vegetables; (5) light-colored vegetables; (6) fruits; (7) soybean and soybean products; (8) milk and dairy products; (9) red meat and products; (10) poultry and game; (11) eggs; (12) fish and shellfish.

Supplementary Table S3. Characteristics of children included and excluded in the present study [a].

	Included (n=1176)	Excluded (n=216)	*P*
**Sex, *n* (%)**			0.437
Male	600 (51.0)	117 (54.2)	
Female	576 (49.0)	99 (45.8)	
**Region, *n* (%)**			0.324
Beijing	406 (34.5)	86 (39.8)	
Changzhi of Shanxi	343 (29.2)	57 (26.4)	
Urumuqi of Xinjiang	427 (36.3)	73 (33.8)	
**Age (year)**	9.61 (0.53)	9.61 (0.55)	0.640
**Anthropometric measures**			
BMI (kg/m^2)	17.94 (5.22)	17.58 (4.66)	0.729
BMI Z-score	0.78 (2.19)	0.67 (1.94)	0.826
WC (cm)	63.25 (14.86)	63.55 (14.31)	0.791
Body fat percentage (%)	19.05 (14.70)	19.35 (14.25)	0.582
**Obesity [b], *n* (%)**			0.480
No	894 (76.0)	169 (78.2)	
Yes	282 (24.0)	47 (21.8)	

[a] Categorical variables were reported as the number (%) and continuous variables were reported as the median (IQR). [b] Obesity was defined using age- and sex-specific BMI percentiles according to the Chinese reference. Abbreviations: BMI, body mass index; WC, waist circumference.

Supplementary Table S4. Effects of intervention on the levels of each subgroup.

	Intervention vs. control Model1 [a]		Intervention vs. control Model2[c]	
	**OR (95%CI)**	***P***	**OR (95%CI)**	***P***
**Under-intake**				
Cereals [b]	1.33 (0.94, 1.87)	0.099	1.34 (0.95, 1.90)	0.092
Vegetable	0.82 (0.54, 1.24)	0.345	0.83 (0.56, 1.25)	0.386
Fruits	0.88 (0.65, 1.18)	0.394	0.91 (0.56, 1.25)	0.507
Milk and dairy products	1.15 (0.81, 1.63)	0.432	1.17 (0.82, 1.66)	0.383
Soybean and soybean products	1.05 (0.76, 1.46)	0.758	1.06 (0.77, 1.47)	0.711
Red meat, poultry and game [b]	1.20 (0.95, 1.53)	0.128	1.20 (0.91, 1.58)	0.199
Fish and shrimp	1.09 (0.71, 1.67)	0.691	1.03 (0.65, 1.64)	0.903
Eggs [b]	1.10 (0.73, 1.65)	0.637	1.12 (0.75, 1.67)	0.920
Drinking water	0.73 (0.52, 1.03)	0.071	0.75 (0.54, 1.05)	0.095
**Over-intake**				
Cereals [b]	0.80 (0.55, 1.16)	0.242	0.78 (0.54, 1.13)	0.186
Red meat, poultry and game [b]	0.95 (0.70, 1.28)	0.719	0.94 (0.68, 1.30)	0.704
Eggs [b]	1.00 (0.64, 1.57)	0.990	0.98 (0.63, 1.52)	0.920
Sugar-sweetened beverage	0.27 (0.19, 0.40)	**<0.001***	0.29 (0.20, 0.41)	**<0.001***
Unhealthy snacks	0.59 (0.37, 0.93)	**0.023***	0.61 (0.39, 0.96)	**0.034***
**Dietary diversity**				
Inadequate dietary diversity	1.00 (0.43, 2.31)	0.991	0.97 (0.39, 2.42)	0.956

[a] Generalized linear mixed model with logistic link function were used, allowing for the school-clustering effect,

with adjustment for age, sex, region, district (urban/suburban) and the dietary outcomes at baseline. [b] Over-intake (score>0) and under-intake (score<0) situations were described for foods both given positive and negative scores in DBI-07.[c] Imputed 15 missing values (lost to follow-up) at the end of the trial using baseline values. [*] *P*<0.05.

Supplementary Table S5. Effects of intervention on three dietary quality indicators.

	Changes from baseline (Intervention vs. control) Model1 [a]		Changes from baseline (Intervention vs. control) Model2 [b]	
	**Adjusted mean difference (95%CI)**	***P***	**Adjusted mean difference (95%CI)**	***P***
**Higher bound scores (HBS)**	-1.53 (-2.41, -0.63)	**0.006**[*]	-1.54 (-2.42, -0.65)	**0.004**[*]
**Lower bound scores (LBS)**	0.02 (-2.13, 2.16)	0.990	0.19 (-2.02, 2.40)	0.873
**Diet quality distance (DQD)**	-1.49 (-2.62, 0.63)	0.217	-1.33 (-3.40, 0.74)	0.259

[a] Linear mixed models were used, allowing for the school-clustering effect, with adjustment for age, sex, region, district (urban/suburban) and the corresponding scores at baseline. [b] Imputed 15 missing values (lost to follow-up) at the end of the trial using baseline values. [*]*P*<0.05.
